# An Examination of the Opinions of NHS Stakeholders on the Current and Future Role of Physician Associates

**DOI:** 10.7759/cureus.96609

**Published:** 2025-11-11

**Authors:** Bashar M Abdeen, Alice Millard, Tareq Al Saoudi, Alex Boddy, Eyad Issa, John Isherwood, Ashley Dennison, Guiseppe Garcea

**Affiliations:** 1 Hepato-Pancreato-Biliary Surgery, University Hospitals of Leicester NHS Trust, Leicester, GBR; 2 Oesophago-Gastric Surgery, University Hospitals of Leicester NHS Trust, Leicester, GBR

**Keywords:** nhs england, nhs workforce, patient opinion, physician associates, physician shortage, quality improvement research

## Abstract

Introduction

Healthcare systems are experiencing problems recruiting and retaining workers and consequently are developing the role of intermediate-level practitioners including Physician Associates (PAs). However, the integration of PAs into the NHS is facing many challenges and there are no studies looking into the causes behind this. This study examines the opinions of NHS stakeholders on the current and future roles of PAs and explores strategies to improve integration.

Methods

This cross-sectional study at a single NHS Trust gathered views and experiences regarding PAs through a survey of consultants, resident doctors (RDs), PAs, and patients. Tailored surveys were generated by doctors and a PA, validated and pilot tested then electronically distributed. Data were analysed using Excel (Microsoft, Redmond, WA, USA) and SPSS (IBM Corp., Armonk, NY, USA) and qualitative analysis for open-ended questions.

Results

Half of the consultants recognised the positive contributions of PAs. However, 68.6% (n=35) felt that PA roles were unclear. The PA role was explained to 11% (n=3) of RDs, 14% (n=4) understood their role, and 75% (n=21) expressed concern that PAs might hinder their training. Amongst the PAs, 75% (n=12) felt the healthcare climate adversely affected their work and 56.3% (n=9) were considering a career change. Only 14% (n=7) of patients fully understood the PA role, but 86% (n=43) were comfortable being seen by one.

Conclusion

This study highlights the contributions of PAs and recognition by stakeholders. Integration, however, remains challenging with concerns regarding RDs' training, and PA career progression and satisfaction. The hurdles are compounded by gaps in understanding the role. Going forwards, clear definition of and education regarding the role of PAs is essential and is the joint responsibility of PAs, managers, consultants, the General Medical Council, and Royal Colleges.

## Introduction

The global shortage of healthcare workers affecting both low and high-income countries including the UK has prompted the examination of alternative models of care [1]. In the UK the result has been an expansion of the Physician’s Associate (PA) role. This expansion has proved remarkably controversial and social media has facilitated the rapid spread of disinformation and served in many instances as a platform for unprofessional dialogue. This resistance is notable given the success of PA programmes in the USA, where PAs have been an established and valued component of healthcare for nearly 60 years, with a 76% increase in their numbers between 2013 (95,583) and 2022 (168,318) [2]. In 2025 it will be 60 years since Dr Eugene Stead at Duke University Hospital, Durham, North Carolina, had the vision to develop a formal programme for PAs as a response to the shortage of clinicians due to increasing specialisation following the Second World War [3]. See the Appendix for milestones in the evolution of Physicians Assistants in the United States [4].

The spiralling cost of healthcare provision is another factor responsible for the adoption by many countries of the model developed in the USA developing healthcare workers with shorter training pathways and broader skill sets [5]. In the UK the roles which are represented are mid-level practitioners (MLPs), which includes PAs and Advanced Nurse Practitioners (ANPs) [6].

In 2003, the UK employed the first PAs from the US to help with the shortages of healthcare workers in primary care in the West Midlands [7]. Two years later, the UK Association of Physician Associates (UKAPA) was established and in 2009, the first cohort of PAs graduated in the UK [8]. The number of PAs steadily increased following these initial deployments and there are presently over 4000 PAs working in primary and secondary care in the NHS. PAs are trained to enable them to be directly supervised by a doctor while working as part of a multidisciplinary team. PAs can assess patients by taking histories, perform physical examinations, request and interpret diagnostic tests, and provide health advice. Their role can evolve with relevant training to perform diagnostic and therapeutic procedures, but in the UK, they are not able to prescribe or request investigations that employ ionizing radiation [9].

The integration of PAs in the NHS has been controversial and in a number of specialties there has been considerable resistance. Issues have manifested in many situations as a difficult working environment for PAs who have different roles and varying levels of support. There also remains a lack of clarity regarding the role of PAs in the NHS partly due to their myriad roles in widely differing specialties in both primary and secondary care. They also presently lack a regulatory body, which raises concerns regarding the scope of practice of PAs and significantly hinders their professional development [10]. 

Additionally, and considering the current negative climate around PAs as seen on X, there is little empirical evidence about the attitudes of key stakeholders - including patients, doctors, and PAs - towards the integration of PAs in the NHS. In this study, we sought the perspectives of stakeholders on the current and future role of PAs within the NHS. The aim was to explore the contributions PAs make to healthcare services, identify the challenges that hinder their integration, and determine what can be done to help them reach their full potential. 

## Materials and methods

Study design and participants

This cross-sectional study, employing a convenience sampling approach across stakeholder groups, was conducted at the University Hospitals of Leicester NHS Trust (UHL) to explore views and experiences related to PAs. Four distinct surveys were developed for consultants, resident doctors, PAs, and patients. The consultant survey targeted all medical and surgical departments across UHL, while the resident doctors’ survey included all doctors below consultant level within the Trust. Patients were randomly selected from those who had interacted with a PA within the four weeks preceding survey distribution. All PAs employed by UHL were invited to participate. Participation in all surveys was voluntary and based on informed consent.

Survey development

The survey items were developed by a multidisciplinary team comprising doctors and a PA. Survey generation was informed by a literature review and interviews with key stakeholders. Each survey was specifically tailored to target its respective group to ensure contextual relevance and appropriateness. Content was validated by a panel of experts, including senior consultants with experience in management and healthcare policy, experienced PAs, and senior resident doctors. Feedback from the panel informed revisions of the surveys to enhance clarity and relevance. Pilot testing was conducted by distributing each survey to five representatives from each stakeholder group to evaluate accessibility and usability, with further adjustments implemented based on their feedback. 

Survey distribution

Surveys were electronically distributed via Microsoft Forms® (Redmond, WA, USA). Consultants, resident doctors, and PAs received their surveys through institutional email, accompanied by weekly reminders over a four-week period to enhance response rates. The patient survey was disseminated through the ACCURX® platform, an NHS-approved communication system. Patients received survey links via text message, accompanied by a brief explanation of the study, with weekly reminders sent for four consecutive weeks.

Data collection and analysis

Data were collected and analysed using Microsoft Excel®. Quantitative data were analysed using SPSS Statistics (IBM Corp., Armonk, NY, USA) software. Chi-square tests were used to compare subgroups across categories. Statistical significance was set at p<0.05. Additionally, 95% confidence intervals were calculated for key proportions to aid interpretation of the findings.

Ethical and institutional approval

This study received approval from the UHL audit and quality improvement programme committee, with registration number 13217. The study was deemed to be low risk and did not require a research ethics committee review. 

## Results

A total of 145 responses were received, consisting of 51 consultants, 28 resident doctors (RDs), 16 physician associates, and 50 patients. Their demographics are presented in Table 1. 

**Table 1:** Demographic characteristics of various survey participants PA: physician associate FY1: foundation year 1 doctor SHO: senior house officer

Participant group	Demographic	Range	Percentage
Patients	Age	18-30	2 (4%)
		31-50	9 (18%)
		51-70	24 (48%)
		70+	15 (30%)
	Gender	Male	30 (66%)
		Female	17 (34%)
	Index of multiple deprivation decile	1-3	11(22%)
		4-6	9 (18%)
		7-10	28 (56%)
		Undetermined	2 (4%)
Physician Associates	Specialty	Medical	10 (63%)
		Surgical	6 (38%)
	Length of time working as a PA (Years)	0-5	12 (75%)
		6+	4 (25%)
Consultants	Time working as a consultant in the NHS	Senior Consultant (>10 years)	22 (43%)
		Junior Consultant (0-10 years)	29 (57%)
	Specialty	Surgical	18 (35%)
		Medical	33 (65%)
	Work directly with a PA	Yes	31 (61%)
		No	20 (39%)
Resident Doctors	Level of training	FY1	8 (29%)
		SHO	10 (36%)
		Registrar	10 (36%)
	Specialty	Medical	16 (57%)
		Surgical	12 (43%)
	Ever worked with a PA	Yes	26 (93%)
		No	2 (7%)

Consultant survey

Patient Care 

**Figure 1:**
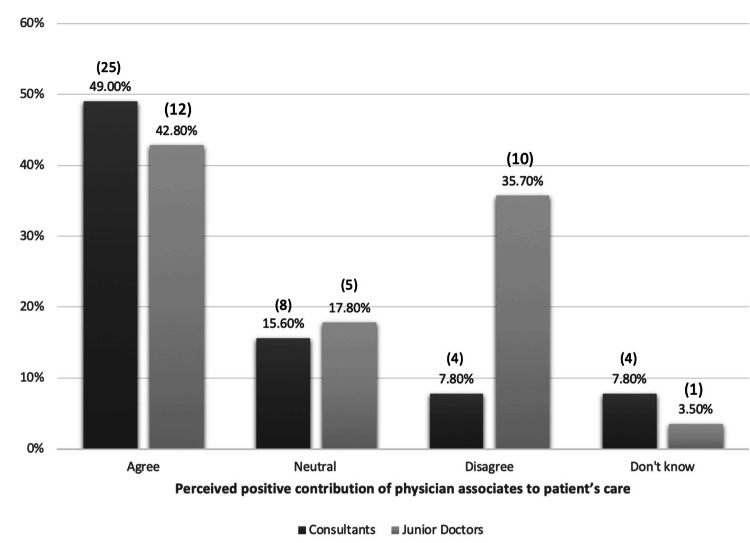
The perceived positive contribution of physician associates to patient care by consultants and resident doctors

Almost half (49%, n=25) of the consultants agreed that PAs positively contribute to patient care. In contrast, 15.6% (n=8) stated PAs have no effect, while 7.8% (n=4) believed PAs impact patient care negatively (Figure 1). Consultants with experience working alongside PAs were more likely to recognize their positive contribution (p=0.012). Consultants were also asked about the role of PAs in clinical settings. A majority (64.7%, n=33) acknowledged that PAs stabilise the team, facilitating smoother transitions particularly during resident doctors' rotations. Additionally, 62.7% (n=32) recognized that PAs enhanced patient care and workflow, while 58.8% (n=30) noted that PAs' helpfulness increases with experience. Moreover, 54.9% (n=28) agreed that PAs ensure continuity of care and provide valuable flexibility during staff shortages. The consultants' specialty, whether medical or surgical, did not significantly influence their views regarding the impact of PAs on patient care.

Role of PAs 

Consultants were queried on their opinions regarding the general roles of PAs. A majority, 68.6% (n=35), noted a lack of clarity regarding PAs' roles, and 72.5% (n=37) agreed that a regulatory authority would enhance these roles. Approximately half believed that PAs would be more effective if they could prescribe medications (47.0%; n=24) and request investigations employing ionising radiation (49%; n=25). 

Resident Doctors and Training 

A quarter of consultants (25.4%; n=13) reported that a trainee had expressed concerns about compromised training opportunities due to the presence of a PA. Senior consultants were more likely to receive such feedback compared to their junior counterparts (p=0.004). Additionally, 50.9% (n=26) believe PAs could alleviate the workload, allowing resident doctors more training opportunities, while 41.1% (n=21) conversely felt that PAs might limit training opportunities for resident doctors. 

Future Role in the NHS 

When queried about the impact of the NHS's plan to increase PA numbers on service levels, 49% of consultants (n=25) anticipated an improvement, 15.6% (n=8) predicted a deterioration, and 7.8% (n=4) believed there would be no significant impact. Those working with PAs were more optimistic about the potential for a positive impact (p=0.006). Among 20 consultants not currently working with PAs, 45% (n=9) saw a potential role for PAs in their departments, while 55% (n=11) did not. Additionally, two-thirds (n=34) expressed concern that the NHS may face challenges in retaining PAs due to a lack of clarity regarding the potential for career progression and the lack of a formal pathway.

In the subgroup analysis, neither the specialty in which consultants worked nor their years of experience significantly influenced their responses. Finally, we posed an open question to respondents about their views on PAs. The three most common themes identified were: 1. PAs positively impact patient care when working within a well-defined scope 2. There is a need for clear definition and regulation of PA roles and responsibilities. 3. Concerns were raised about a potential reduction in training opportunities for resident doctors.

Resident doctors’ survey 

Understanding the Role of PAs

**Figure 2:**
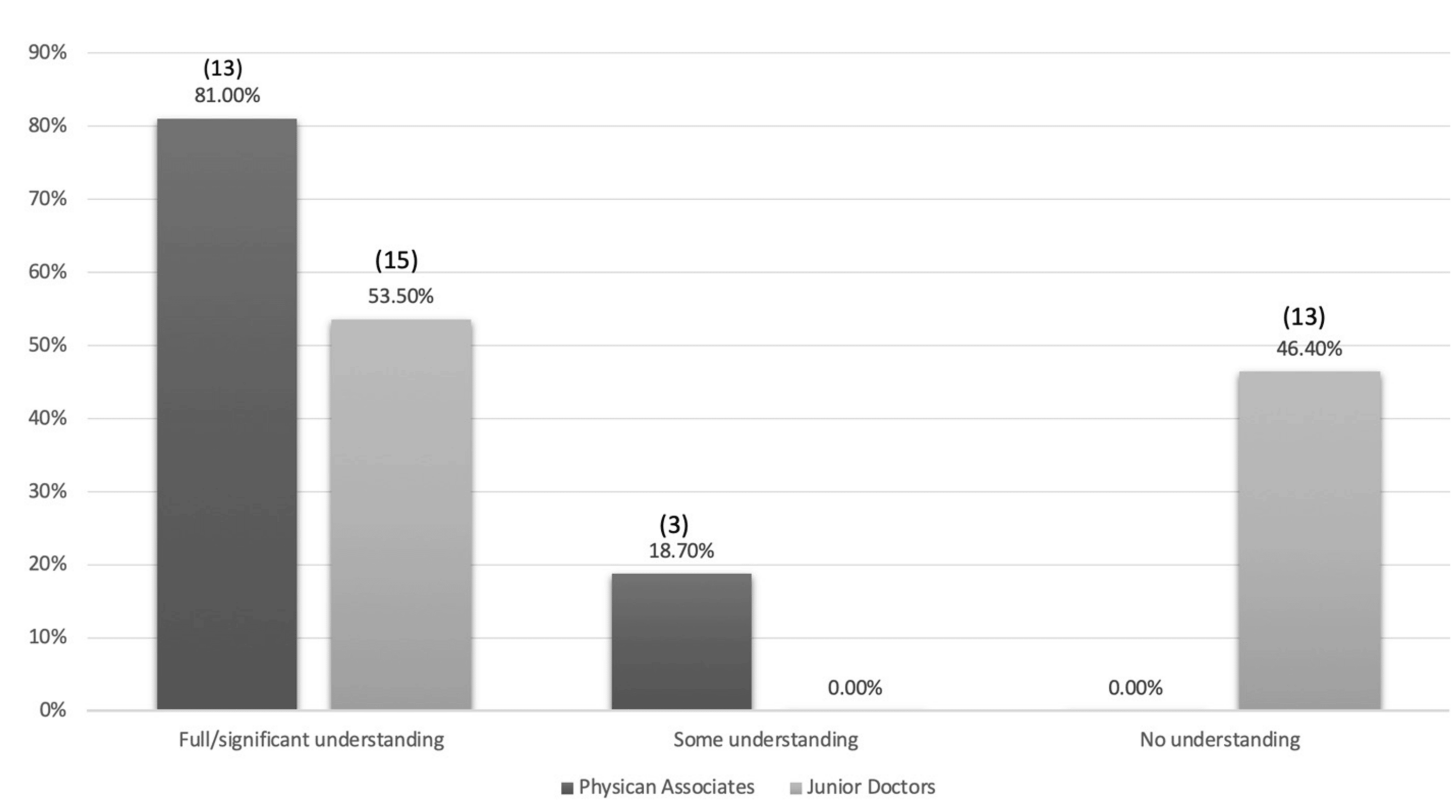
Comparison of resident doctors’ understanding of the physician associates’ role vs. physician associates’ assumptions of resident doctors’ understanding

Of the 26 resident doctors who worked with a PA, only 11% (n=3) reported that the PA's role was explained to them during induction, either by a consultant or the PA themselves, and all three confirmed that the explanation was clear. In a separate query about their understanding of PAs' roles in healthcare, 14% (n=4) of resident doctors stated that they fully understood it, 39% (n=11) had a partial understanding, and 46% (n=13) had little to no understanding (Figure 2). Furthermore, 82% (n=23) acknowledged a lack of clarity regarding the role of PAs. 

Patient Care 

Of the resident doctors surveyed, 43% (n=12) agreed that PAs contribute positively to patient care, while 36% (n=10) disagreed with this statement. Eighteen percent (n=5) believed that PAs do not make any difference (Figure 1).

Effect on Resident Doctors’ Workload 

When resident doctors were asked about the impact of working with a PA on their workload, responses were divided: 38% (n=10) said it decreased their workload, another 38% (n=10) felt it increased, and 23% (n=6) noticed no difference. Additionally, regarding their willingness to prescribe medication based on a PA's assessment and recommendation, 64% (n=18) were not comfortable doing so without their own assessment. Having a good working relationship was felt to be a significant factor in prescribing medication on advice from a PA by 36% (n=10), whilst 25% (n=7) said their decision would depend on the medication involved, and 18% (n=5) would consider it if it was a consultant's decision. 

Resident Doctors' Training 

With regards to training opportunities, 75% (n=21) of resident doctors felt that the presence of PAs might negatively affect their training. Conversely, only 7% (n=2) believed PAs improve their opportunities and 18% (n=5) believed there was no impact. When resident doctors with experience working alongside PAs when queried, 23% (n=6) reported some positive impact on their training from this interaction, although 54% (n=14) still perceived a negative impact, and 23% (n=6) noted no impact at all. 

Future Role in the NHS 

When asked about the impact of formal regulation on the role of PAs, 71% (n=20) said it would improve patient safety, and 64% (n=18) felt it would allow for fitness investigations when necessary. Seven (25%) indicated that such regulation would enable PAs to enrol in prescribing courses and clarify their scope of work. Approximately two-thirds (64%, n=18) expressed concern that the NHS plan to increase the number of PAs could hinder their future careers, 14% (n=4) believed it would improve their work environment and 7% (n=2) saw no impact at all. Doctors with prior experience working with PAs were more likely to perceive potential negative effects (p=0.027). Additionally, 25% (n=7) and 18% (n=5) of doctors believed that expanding PAs' roles to include prescribing and requesting ionising radiation respectively, would be beneficial. 

Finally, we posed an open question to resident doctors about their views on PAs. The responses revealed three main themes. Many noted that PAs could alleviate resident doctor workload by handling routine tasks and responsibilities. Secondly, there were concerns about training opportunities specifically how competition between PAs and resident doctors for limited opportunities might reduce the available training opportunities. Finally, some respondents emphasized that PAs should be strictly regulated and always supervised to prevent potential harm to patients.

Physician associates survey 

Results from the survey of 16 PAs were analysed, with their demographics presented in Table 1. Additionally, 11 of the 16 PAs reported working alongside other PAs within their departments.

Perceived Understanding of the Role

When surveyed about how well their colleagues understand their roles, the majority of PAs felt that both consultants (87.5%, n=14) and resident doctors (81.3%, n=13) were aware of their responsibilities (Figure 2). A smaller proportion (62.5%, n=10) believed that the broader healthcare team, including nurses and allied health professionals, understood their role well. All PAs surveyed felt that their clinical teams were aware of and supportive of the limitations associated with their roles. Half of the PAs (50%, n=8) felt that their patients understood their role, 18.8% (n=3) believed patients did not fully understand and the remainder were neutral. Despite these perceptions, one-third of PAs acknowledged that there remained some ambiguity surrounding their role.

Current Role

Considering the constraints faced by PAs within the current healthcare climate, participants were asked about perceived support within their departments. The majority (93.7% n=15) reported feeling supported by consultants and resident doctors and all expressed having a positive relationship with the doctors on their teams. Furthermore, all PAs reported receiving adequate supervision in their roles. 

Future Role in the NHS 

In terms of future prospects and career trajectory, 62.5% (n=10) believed in the potential for career progression and 87.5% (n=14) were satisfied with their current training opportunities. Five (31.25%) expressed concerns about the NHS’s ability to retain PAs under the presently prevailing conditions, with 56.3% (n=9) considering a career change or relocation. The majority indicated that the current healthcare climate was adversely affecting their work (75%, n=12) and mental well-being (93.8% n=15), though only two (12.5%) PAs experienced workplace hostility. A significant number (86.7% n=14) believed that regulatory changes would benefit their role. Respondents also saw enhanced opportunities for prescribing (86.7%, n=14) and requesting ionising radiation (80%, n=13) as beneficial additions to their role. Regarding the impact on resident doctors' training, over two-thirds of PAs felt their role could enhance training opportunities in the long term, while only one (6.7%) believed it would be detrimental. 

The analysis of the effect of specialty on survey responses found no significant associations. The open question at the survey's conclusion allowed respondents to offer additional comments. Thematic analysis of these responses highlighted a common desire among PAs for professional recognition, regulatory reforms, improved training, and expanded clinical roles. Respondents also expressed concerns about the challenges posed by perceptions on social media. 

Patients survey 

Out of 141 patients invited to the survey, 50 responded, all of whom had encountered a PA in an outpatient setting (see details in Table 1). 

Term and Role Understanding 

**Figure 3:**
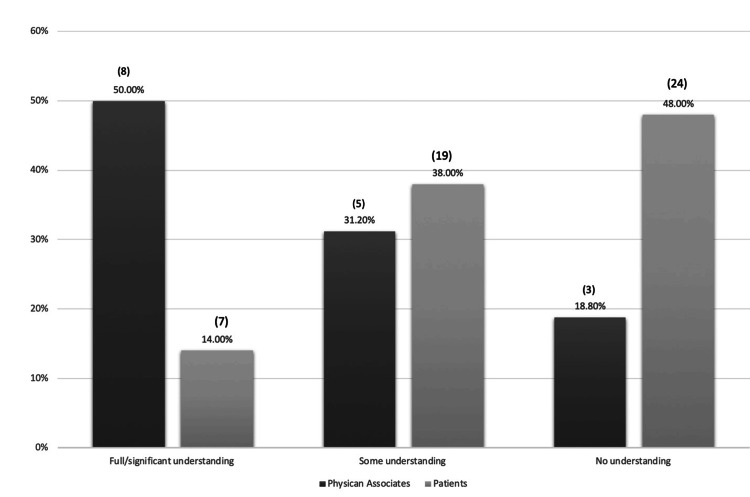
Comparison of Patients’ understanding of the Physician Associates' role vs. Physician Associates' assumptions of Patients’ understanding

Twenty-two patients (44%) were familiar with the term 'Physician Associate,' while the remainder had little or no understanding. Only 14% (n=7) claimed a full understanding of the role, with most indicating some (38%, n=19) or no (48%, n=24) understanding (Figure 3). When asked about the tasks PAs perform, 40% (n=20) felt they were not sufficiently informed to comment. Similarly, 58% (n=29) were unaware of PA training pathways. Although all patients were identified from clinic letters as having been seen by a PA, only 30% (n=15) confirmed such encounters during their care and 28% (n=14) were uncertain. Regarding introductions, half of the patients could not recall how the PA introduced themselves during the clinic visit, with only 20% (n=10) remembering an introduction as part of the medical team or as a Physician Associate. Encounter settings showed that 64% (n=32) were seen in outpatient settings, equally divided between initial consultations and follow-up appointments. Perceived understanding of the PA role did not significantly influence responses to subsequent survey questions.

Patients’ Acceptance and Satisfaction 

A substantial majority of patients (86%, n=43) felt comfortable being seen by a PA in a medical setting. Additionally, 72% (n=36) were comfortable having minor procedures performed by a PA, and 74% (n=37) were at ease with a PA assisting during surgery. Seventy-six percent (n=38) appreciated the possibility of a PA speeding up their appointment times. Regarding prescription authority, 28% (n=14) were unsure, 62% (n=31) supported prescribing rights for PAs with specialised training, and 10% (n=5) were opposed. Notably, patients who recalled being seen by a PA were significantly more likely to agree that PAs should have prescribing privileges (p=0.03) and reported higher satisfaction levels (p<0.001). 

Patients were asked to reflect on their encounters with PAs and rate their satisfaction. None expressed dissatisfaction and 66% (n=33) reported being somewhat or very satisfied. No patient viewed PAs as negatively affecting the NHS; 76% (n=38) recognized their positive contribution, while the rest remained neutral. Awareness of PAs was significantly linked to patients' understanding of the role (p<0.001) and their satisfaction (p=0.026). Satisfaction with the PA experience was significant across various aspects, including comfort with PAs performing minor procedures, assistance during surgery, expedited appointment times, prescribing practices, and perceived impact on the NHS (p<0.001, p<0.001, p=0.006, p<0.001, and p=0.009, respectively) (Table 2). Notably, patients' age, gender, and socioeconomic status, measured by the index of multiple deprivation, showed no significant associations with responses to the survey questions.

**Table 2:** Association Between Awareness, Satisfaction, and Patient Perceptions of Physician Associates (PAs)

Relationship Tested	Crosstab Counts (by Group)		p-value
Awareness (Heard of PA) ↔ Understanding role	Heard=Yes	Understand role=22	<0.001
		Neutral= 6	
		Do not understant= 0	
	Heard=No	Understand role=13	
		Neutral=2	
		Do not understand=7	
Awareness (Heard of PA) ↔ Satisfaction	Heard=Yes	Unsatisifed=8	0.026
		Neutral=6	
		Satisfied=14	
	Heard=No	Unsatisifed=2	
		Neutral=1	
		Satisifed=19	
Satisfaction ↔ Comfort with PAs performing minor procedures	Unsatisfied=10		<0.001
	Neutral=7		
	Satisfied=33		
Satisfaction ↔ Assistance during surgery	Unsatisfied=10		<0.001
	Neutral=7		
	Satisfied=33		
Satisfaction ↔ Expedited appointment times	Unsatisfied=10		0.006
	Neutral=7		
	Satisfied=33		
Satisfaction ↔ Prescribing practices	Unsatisfied=10		<0.001
	Neutral=7		
	Satisfied=33		
Satisfaction ↔ Positive contribution to NHS	Unsatisfied=10		0.009
	Neutral=7		
	Satisfied=33		

The open question in the survey elicited a range of views on PAs in the NHS. Thematic analysis highlighted some uncertainty about the PA role. However, it also revealed recognition of PAs' positive impact and a call for more information and better integration within healthcare teams.

## Discussion

This study revealed a shared recognition among consultants, RDs, PAs, and patients that PAs make a valuable contribution to multidisciplinary healthcare teams. However, it also highlighted persistent uncertainties about the PA role, limited understanding among some stakeholder groups, and structural barriers that hinder full integration within the NHS workforce.

Overall, consultants and RDs acknowledged the stabilising influence of PAs in clinical teams. PAs were recognised for their adaptability, continuity of care, and capacity to reduce service pressures, particularly given the rotational nature of RD training. Similarly, most patients expressed confidence in being assessed or treated by a PA, reflecting the profession’s growing acceptance among the public.

Despite the general agreement regarding the positive contributions of PAs, it remains difficult to integrate this new role into clinical teams [11]. Ritsema et al. found that the main factor facilitating the integration of PAs in the NHS is clearly defining their role. PAs need to understand their role clearly and be able to articulate it to their colleagues and patients. A clearer understanding of the PA role fosters trust among colleagues, strengthens interprofessional collaboration, and allows all team members to work to the top of their scope [12]. Patients also reported dissatisfaction when the role of their care provider was not explained clearly [8]. This lack of role definition undermines PA job satisfaction and makes them vulnerable to potential exploitation, particularly being asked to perform tasks beyond their skill set and remit [6]. In our study, consultants who have worked with a PA were more likely to recognise their positive contributions to patient care, and patients who recall their interaction with a PA are more likely to have a favourable opinion.

Nevertheless, the study also exposed a marked deficit in understanding of the PA role, especially among RDs and patients. PAs themselves tended to overestimate how well their role was understood by others. This finding mirrors previous NHS-based research suggesting that insufficient education and communication remain central barriers to PA integration [10-12]. Only 10% of RDs in our cohort reported that the PA role was explained during induction, indicating the need for formalised role briefings. Patients also reported dissatisfaction when the role of their care provider was not explained clearly [8]. Ritsema et al. found that the main factor facilitating the integration of PAs in the NHS is clearly defining their role. PAs need to understand their role clearly and be able to articulate it to their colleagues and patients. Better understanding of the PA role increases trust between them and their healthcare colleagues, improving work relationships and maximising everyone’s roles [12]. All this evidence highlights the need for clear role definition.

Despite the General Medical Council (GMC) being the regulator, it has stated that it will not set a scope of practice for PAs, as it does not do so for other professions. The GMC said the responsibility to define the scope of work falls on local policy and individual PA skills, supporting relevant organisations to set the scope of practice beyond certification [13]. The authors believe that this responsibility falls on the Faculty of Physician Associates and the Royal Colleges who should define the scope of work for newly qualified PAs and establish a clear training route. Local authorities must also define a clear role and training pathway and communicate this to different stakeholders.

Concerns about the potential impact of PAs on RD training were also prominent. Three-quarters of RDs expressed apprehension that PAs might limit their training opportunities, echoing findings from the Association of Surgeons in Training (ASiT) [14]. In the ASiT survey, 70% of respondents said PAs had a negative impact on their surgical training. Resident doctors at lower training levels (Foundation, CSTs) were more likely to report a negative effect on their training than higher-level trainees [14]. Consultants were divided, with some noting that PAs can relieve workload, indirectly enhancing training time, while others perceived them as competitors for clinical exposure. This perception may stem from consultants’ preference for established team members, combined with the immense pressures on the NHS caused by waiting lists and staff shortages. The current expansion of PAs across the NHS has further narrowed training opportunities. These mixed perceptions underline the need for clear guidance from the GMC and Royal Colleges on the training and supervision of RDs by consultants, and how PAs can facilitate patient care and progress in their careers without negatively affecting RDs. However, local hospital guidance and consultant attitudes in prioritizing training are of paramount importance, especially with the GMC's proposed policy of delegating the definition of roles to local hospitals.

The survey also revealed growing unease among PAs about their professional identity and career security. Many respondents reported uncertainty regarding their future within the NHS, citing the lack of regulation, unclear scope of practice, limited progression routes, and a hostile online discourse. This is prompting a significant proportion of PAs to consider alternative careers or relocation to other countries. Contributing to these concerns are the recent resignations of numerous members of the Faculty of Physician Associates [15], upcoming changes in GMC regulation with unclear impacts [16], a lack of job opportunities [17], and a hostile atmosphere both in the workplace and on social media [18]. These socio-political and organisational factors may undermine morale and retention, posing risks to the sustainability of the workforce. Until these questions are addressed, it is crucial that PAs are perceived as working collaboratively to support doctors, not as their replacements. Enhancing the understanding and awareness of the PA role is imperative to ensure that PAs are held accountable and work within their defined scope of practice. This responsibility, again, falls on the national and local regulating bodies. 

Taken together, these findings reinforce that the future of the PA role depends on clarity, communication, and collaboration. National and local leadership must prioritise defining responsibilities, establishing structured governance, and fostering interprofessional trust to fully realise the contribution of PAs to NHS sustainability.

This study has some limitations. Data were collected from a single NHS Trust, with a relatively small sample, which may limit generalisability. Surveys were conducted during a period of heightened tension between PAs and resident doctors, which could have influenced responses and biased results. Future research should include larger, more diverse samples across multiple Trusts and examine departmental performance before and after hiring PAs to provide objective measures of their impact.

## Conclusions

In conclusion, this study underscores that PAs make a valuable contribution to NHS clinical teams, improving continuity and supporting service delivery. However, their successful integration remains constrained by unclear role definitions, limited understanding among colleagues and patients, and concerns about their impact on medical training. Sustainable progress requires a coordinated approach involving the GMC, Royal Colleges, local trusts, and educational institutions. This should include the establishment of a defined scope of practice, transparent supervision frameworks, and structured career pathways. Clearer communication and role education are also essential to strengthen collaboration, ensure accountability, and improve public and professional confidence in this emerging workforce. By addressing these organisational and cultural challenges, the NHS can harness the full potential of PAs to enhance patient care, workforce resilience, and service efficiency. The NHS may also draw lessons from the successful integration of PAs in other healthcare systems, such as those in the USA, Canada, and the Netherlands.

## Appendices

**Table 3:** Milestones in the evolution of physicians assistants (PAs) in the USA modified from the PA Profession Historical Milestones: aapa.org [4]

Date	Development	Significance
1961	Proposal for “an advanced medical assistant with special training, intermediate between that of the technician and that of the doctor,	The World Health Organization introduces and promotes health care workers in developing countries (Medecin Africain, dresser, assistant medical officer, rural health technician, etc.)
1965	Eugene A. Stead Jr., MD, establishes a “physician assistant” educational program at Duke University in North Carolina.	Program accepts four former Navy medical corpsmen (Kenneth F. Ferrell, Victor Hugo Germino, Jr., Richard John Scheele, Donald Guffey).
1967	The first PA class graduates from the Duke University PA program	October 6 1967
1968	The American Association of Physician Assistants is incorporated.	AAPA become the American Academy of PAs
1971	The American Medical Association (AMA) recognizes the PA profession.	The first six states pass legislation authorizing PA practice
1973	First national PA certifying examination	Administered by the National Board of Medical Examiners (NBME)
1977	PAs in certified rural health clinics begin receiving Medicare reimbursement.	
1987	National PA Day (October 6, 1987) is celebrated for the first time to honour the 20th anniversary of the first graduating class of PAs.	AAPA celebrates PA Week every October 6-12 to recognize the PA profession and its contributions to the nation’s health
1992	PAs are commissioned in every branch of the U.S. military.	
1997	PAs are recognized as Medicare covered providers	Recognised in all clinical areas
2000	Mississippi passes legislation to recognise PAs	All 50 States authorise PA practice
2007	Indiana passes legislation authorizing prescribing by PAs.	PAs obtain prescriptive authority in all 50 states
2008	AAPA identifies Six Key Elements of a Modern PA Practice Act.	Rhode Island is the first state to adopt all the elements.
2010	PAs named in the Patient Protection and Affordable Care Act (ACA) as one of three healthcare professionals.	Included with physicians and nurse practitioners as providers of primary care in the United States.
2016	Ohio adopts use of the term “licensure,” the highest recognized standard for medical providers.	First of AAPA’s Six Key Elements of Modern PA Practice is adopted in all 50 states
2017	Fiftieth anniversary of the profession:	AAPA House of Delegates passes Optimal Team Practice, a new policy that will make a profound difference in expanding access to care and aligning the PA profession to meet modern healthcare needs.
2019	North Dakota becomes first state to enable nearly all PAs in the state to practice without a specific relationship with a physician.	A significant development which was followed by an increase in applications for the PA programmes
